# Fatal Case of West Nile Neuroinvasive Disease in Bulgaria

**DOI:** 10.3201/eid2212.151968

**Published:** 2016-12

**Authors:** Magdalena Baymakova, Iva Trifonova, Elitsa Panayotova, Severina Dakova, Monia Pacenti, Luisa Barzon, Enrico Lavezzo, Yancho Hristov, Konstantin Ramshev, Kamen Plochev, Giorgio Palu, Iva Christova

**Affiliations:** Military Medical Academy, Sofia, Bulgaria (M. Baymakova, S. Dakova, Y. Hristov, K. Ramshev, K. Plochev);; National Center of Infectious and Parasitic Diseases, Sofia (I. Trifonova, E. Panayotova, I. Christova);; Padova University Hospital, Padova, Italy (M. Pacenti);; University of Padova, Padova (L. Barzon, E. Lavezzo, G. Palu)

**Keywords:** West Nile neuroinvasive disease, West Nile virus, flavivirus, viruses, Central/Southern European West Nile virus lineage 2, vector-borne infections, zoonoses, Bulgaria

**To the Editor:** West Nile virus (WNV) is a mosquitoborne flavivirus. Approximately 80% of human infections are asymptomatic, 10%–20% are characterized by an acute febrile illness, and <1% by involvement of the central nervous system (West Nile neuroinvasive disease) (*1*). Sporadic human cases and small outbreaks of West Nile fever were reported in Europe until the mid-1990s (*2*), when the first large outbreak occurred in Romania in 1996 (*3*).

Since then, and especially in recent years, sporadic human cases and outbreaks have been reported in other countries in Europe and neighboring countries on the Balkan Peninsula (*2*). A large outbreak of WNV lineage 2 infection occurred in Greece in 2010 (*4*). Outbreaks have also been reported in other countries in Europe, which showed spread of WNV lineage 2 (*5**–**8*). Some probable cases of West Nile fever were reported to the Bulgarian Ministry of Health on the basic of serologic test results.

We report a case of fatal West Nile neuroinvasive disease in a man in Bulgaria. This case was confirmed by detection of specific antibodies against WNV and sequencing of the full virus genome.

A 69-year-old man was admitted to the Emergency Center, Military Medical Academy (Sofia, Bulgaria), on August 27, 2015, because of fever, headache, hand tremor, muscle weakness and disability of lower extremities, nausea, and vomiting. These signs and symptoms developed 3 days before hospitalization. The patient reported being bitten by insects through the summer. He also had concomitant cardiovascular disease. In the 24-hour period after hospitalization, a consciousness disorder and deterioration of the extremities’ weakness developed, and the patient had a Glasgow come score <8.

The patient was transferred to Department of Intensive Care. Neurologic examination showed neck stiffness, positive bilateral symptoms of Kernig and Brudzinski, right facial paralysis, and areflexia of the lower extremities. The patient underwent intubation, and despite complex medical therapy, a cardiopulmonary disorder developed, and he died 14 days after admission.

Laboratory test results at admission were within reference ranges. Lumbar puncture was performed, and cerebrospinal fluid (CSF) testing showed a clear color, leukocytes 39 ×10^6^ cells/L (reference range 0–5 ×10^6^ cells/L), polymorphonucleocytes 2% (0%–6%), lymphocytes 93% (40%–80%), monocytes 5% (15%–45%), protein 0.57 g/L (0.2–0.45 g/L), glucose 4.3 mmol/L (2.2–3.9 mmol/L), and chloride 127.9 mmol/L (98–106 mmol/L).

Microbiological investigations of blood, CSF, urine, and throat swab specimens showed no bacterial growth. Immunoserologic test results for neurotropic infectious and parasitologic agents were negative, except for a positive result for IgM against WNV. On the basis of these findings, CSF and urine samples were sent to Bulgarian Reference Laboratory of Vector-Borne Pathogens (Sofia, Bulgaria) for confirmation.

Results of serum and CSF tests (WNV ELISA; EUROIMMUN, Lübeck, Germany) were positive for WNV IgM and negative for WNV IgG. A second serum sample obtained 7 days later showed a marked increase in WNV IgM titer and positive results for WNV IgG. WNV RNA was detected by using real-time reverse transcription PCR (Sacace Biotechnologies, Como, Italy) (cycle threshold 21.9) with a urine sample. Blood samples showed negative results for WNV RNA.

Sequencing of the complete genome of WNV obtained from a urine sample (*9*) was performed (GenBank accession no. KU206781). Phylogenetic analysis showed that the virus belonged to the Central/Southern-European WNV lineage 2 clade and the Greek cluster (*6*). Sequence showed high similarity with Greece Nea Santa 2010 and Hungary/578 strains (99.66% and 99.57% nt identity, respectively), which suggested that the virus probably had a common ancestor with Greek strains.

Accordingly, analysis of the polyprotein identified amino acid substitutions that are typically found in WNV strains from Greece (i.e., NS2B V119I, NS3 H249P, NS4B S14G/T49A/V113M) (*6*) and unique mutations not present in other strains (i.e., E I159M, T436A, NS1 K92N, NS4B N220D, NS5 D141G). These results indicate that the virus might have evolved independently before its emergence in Bulgaria.

European lineage 2 of WNV was detected in Hungary in 2004 (*10*). After its introduction into central Europe, this lineage has spread to neighboring countries (*2*), where it has been responsible for several human outbreaks of neuroinvasive disease associated with a high mortality rate, especially in persons with concurrent illnesses (*8*), such as the patient in this study.

This case of WNV infection provides evidence of WNV lineage 2 circulation in Bulgaria and confirms spread of this lineage in Europe. Sequencing of the complete WNV genome enables us to obtain evidence for the possible origin of the Bulgarian strain from WNV strains circulating in Central Europe, from which the Greek strain has also evolved (*4**,**6*). On the basis of this evidence of WNV circulation in Bulgaria, public health institutions should increase WNV surveillance and control programs in the country. Physicians should also actively search for West Nile fever in patients with acute febrile syndrome during the season of mosquito activity.
